# Current use of medical eponyms – a need for global uniformity in scientific publications

**DOI:** 10.1186/1471-2288-9-18

**Published:** 2009-03-09

**Authors:** Narayan Jana, Sukumar Barik, Nalini Arora

**Affiliations:** 1 Department of Obstetrics and Gynaecology, Institute of Postgraduate Medical Education and Research, Kolkata 700020, India; 2 Department of Obstetrics and Gynaecology, Westbank Hospital, Howrah 711109, India

## Abstract

**Background:**

Although eponyms are widely used in medicine, they arbitrarily alternate between the possessive and nonpossessive forms. As very little is known regarding extent and distribution of this variation, the present study was planned to assess current use of eponymous term taking "Down syndrome" and "Down's syndrome" as an example.

**Methods:**

This study was carried out in two phases – first phase in 1998 and second phase in 2008. In the first phase, we manually searched the terms "Down syndrome" and "Down's syndrome" in the indexes of 70 medical books, and 46 medical journals. In second phase, we performed PubMed search with both the terms, followed by text-word search for the same.

**Results:**

In the first phase, there was an overall tilt towards possessive form – 62(53.4%) "Down's syndrome" versus 54(46.6%) "Down syndrome." However, the American publications preferred the nonpossesive form when compared with their European counterpart (40/50 versus 14/66; P < 0.001). In the second phase, PubMed search showed, compared to "Down syndrome," term "Down's syndrome" yielded approximately 5% more articles. The text-word search of both forms between January 1970 and June 2008 showed a gradual shift from "Down's syndrome" to "Down syndrome," and over the last 20 years, the frequency of the former was approximately halved (33.7% versus 16.5%; P < 0.001). The abstracts having possessive form were mostly published from the European countries, while most American publications used nonpossesive form consistently.

**Conclusion:**

Inconsistency in the use of medical eponyms remains a major problem in literature search. Because of linguistic simplicity and technical advantages, the nonpossessive form should be used uniformly worldwide.

## Background

Eponyms are in daily use in medicine. Eponym indicates the name of a person after whom something such as a discovery, invention, institution etc is named usually to commemorate the importance of his/her contribution. A recent debate entitled "Should eponyms be abandoned?" evoked strong responses both in favour and against the motion, and added interesting insights to the current use of medical eponyms [1,2]. The proponents call on the editors of medical journals and textbooks to abandon the use of eponyms because they "lack accuracy, lead to confusion, and hamper scientific discussion in a globalised world" [1]. On the contrary, the opponent supports retention of eponyms, as they are "often practical and a form of medical shorthand," and "bring colour to medicine and they embed medical traditions and culture in our history" [2]. Nevertheless, the use of medical eponyms, as in other areas, is often random, inconsistent, idiosyncratic, confusing, and even misleading [1-4]. One common inconsistency is, use of same eponym in its possessive form (eg, Down's syndrome) as well as nonpossessive form (eg, Down syndrome), which hampers retrieval of information from a public databases [3,5,6]. This variation is arbitrary, not governed by rule [3]. Although the problem was identified more than 30 years ago when an international working group clearly recommended discontinuation of all possessive forms [7,8], the problem still remains unresolved [3,5,6,9-13]. Therefore, the present study was planned to assess the current use of medical eponyms in its nonpossessive versus possessive form taking "Down syndrome" and "Down's syndrome" as an example, and to explore any changing trend. Furthermore, we have attempted to identify and bridge the gaps between the existing recommendations, and current state of its implementation regarding use of the eponymous terms.

## Methods and results

This study was carried out in two phases – first phase in 1998 and second phase in 2008. In the first phase, we searched manually the terms "Down syndrome" and "Down's syndrome" in the indexes of 70 medical books published during 1990–97, and the annual indexes (1996–97) of 50 medical journals related to obstetrics, paediatrics, neonatology, radiology, genetics and general medicine. Statistical analysis included χ^2 ^test with Yates correction. The overwhelming number of publications (116 of 120) were either American (the United States) or European (mostly from the United Kingdom). Therefore, only those were included for comparison (Table 1), which showed a tilt towards possessive form – 62(53.4%) "Down's syndrome" versus 54(46.6%) "Down syndrome." It was also revealed that American publications preferred the latter when compared with their European counterpart (40/50 versus 14/66; P < 0.001); this was consistent both for the textbooks and journals.

**Table 1:** "Down syndrome" and "Down's syndrome" in 116 medical publications.

	American publications (n = 50)		European publications (n = 66)		*P value
			
	Down syndrome	Down's syndrome	Down syndrome	Down's syndrome	
Journal indexes	19(86.4)	3(13.6)	5(20.8)	19(79.2)	P < 0.001
Textbook indexes	21(75)	7(25)	9(21.4)	33(78.6)	P < 0.001
Both indexes	40(80)	10(20)	14(21.2)	52(78.8)	P < 0.001

Values are numbers (percentages). This table excluded 2 Asian and 2 Australian publications.

* χ^2 ^test with Yates' correction

In the second phase of study, we searched PubMed database several occasions (August 23, 2005; January 19, 2007 and June 19, 2008) with the phrase "Down syndrome" and "Down's syndrome" and retrieved a consistently different number of studies (Table 2). Compared to "Down syndrome," terms "Down's syndrome" and "trisomy 21" yielded approximately 5% and 10% more articles, respectively. Considering this difference, we selected first 200 abstracts retrieved from PubMed search (accessed June 19, 2008) with the term "Down syndrome" and then performed a text-word search for "Down syndrome" and "Down's syndrome" in these abstracts along with the titles. Only 63 abstracts contained either of these terminologies – 55(87.3%) with "Down syndrome," and 8(12.7%) "Down's syndrome;" and one abstract contained both the terms [14]! Those abstracts which used the possessive form were mostly published from European countries. In contrast, most American journals used nonpossesive form more consistently.

**Table 2:** Number of articles retrieved following PubMed search with the terms (not text-word) "Down syndrome" and "Down's syndrome."

Date of search	Number of articles with the term "Down syndrome"	Number of articles with the term "Down's syndrome"
June 19, 2008	21,988	23,191
Within the last 5 years	4,483	4,718
Within the last 1 year	950	1,022
January 19, 2007	17,497	17,592
August 23, 2005	15,462	15,714

Difference between 2 groups over the time period is not significant (p > 0.05).

**Figure 1:**
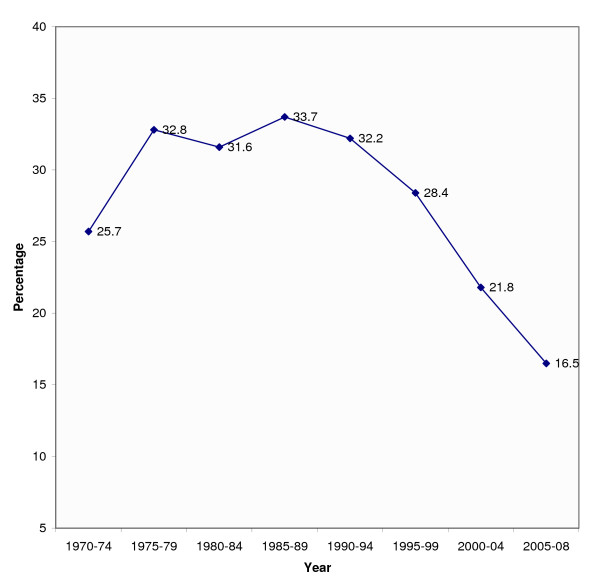
**Trend in use of "Down's syndrome" versus "Down syndrome"**. Trend in use of possessive form i.e., "Down's syndrome" in PubMed archives since 1970. Percentage is calculated using a formula, 100X/(X+Y), where X and Y indicated the total number of articles retrieved by text-word search "Down's syndrome" and "Down syndrome," respectively. Over the last 20 years, this frequency is approximately halved (33.7% versus 16.5%; P < 0.001).

To explore the trend, we performed text-word search of both forms in the PubMed for a consecutive 5-year block from 1970–74 to 2005–08 (June 30). There was a gradual shift from "Down's syndrome" to "Down syndrome." From 1990, there has been a steady decline (4–6% per every 5-year) in the usage of possessive form (Figure 1), and over the last 20 years, this frequency is approximately halved (33.7% versus 16.5%; P < 0.001).

The above findings prompted us additional studies on the use of eponyms in relation to other diseases – Alzheimer disease and Parkinson disease. PubMed search revealed indiscriminate use of possessive and nonpossessive forms for both the eponymous diseases, although their MeSH terms are in nonpossessive form. Furthermore, search of commonly used resources on the internet also revealed use of both forms of each eponym. There were wide differences in the number of hits with search phrase "Down syndrome" and "Down's syndrome" in the websites such as Google or Yahoo. Similar results were also obtained for possessive and nonpossessive forms of Alzheimer disease and Parkinson disease in these websites.

## Discussion

Appropriate and uniform use of nomenclature of a clinical disorder is vital for its identification, classification, and retrieval of information from a public database [6]. The aim of this uniformity is to provide a flexible and practical, yet scientifically acceptable term to describe an abnormality [8]. Although no one would dispute the desirability of a disease name that relates directly to its basic defects and aetiology [5], this would be exceedingly difficult in many conditions where such information is yet to be known [13]. Use of eponyms in medicine and other branches of science is widespread [3]. Over the years many people have condemned use of medical eponym [1,6,15], while the others have found it useful, practical, and even interesting [2,15]. As total elimination is an unpragmatic task [6], it is immensely important that they are recorded, indexed and retrieved uniformly [16]. The purpose of this study is to clear the path towards uniformity by casting light upon the technical and practical use of medical eponyms. The linguistic terrain has been explored earlier [3]. Our study highlights the variation of a common eponym in medical publications, and this has seriously impeded information retrieval from electronic archives. Use of standardised terminology promotes communication, facilitates spell-checking and avoids confusion both in basic and clinical sciences [16,17].

In this study, Down syndrome has been used as an example because of several reasons – it is one of the long-lived eponyms used worldwide without many variations; its both possessive and nonpossesive forms are widely prevalent within a country, journal, textbook, monograph, or even within an article; it has dropped an old eponym that has "misleading racial connotation" ie, Mongolism [18], and it has also acquired a descriptive name, trisomy 21. Therefore, this eponym encapsulates many diverse facets of eponymous medical terms – a colourful history punctuated by controversy and debate, a credible scientific understanding regarding its aetiology that has ushered an alternative name, and also reflects flexibility of medical community towards acceptance of an eponymous nomenclature.

Additional search in common internet resources (Google and Yahoo) with 2 other eponyms (Alzheimer disease and Parkinson disease) also reveals that this problem is neither unique to Down syndrome nor to PubMed, but is ubiquitous, affecting search results for many eponymous terms from wide range of electronic databases. Wikipedia [19], a free on-line encyclopedia prefers "Down syndrome" as main heading, while it includes possessive form for many other eponyms (eg, Alzheimer's disease and Parkinson's disease). Most patients' websites prefer use of nonpossessive form [eg, European Down Syndrome Association, National Association for Down Syndrome (U.S.A.), Canadian Down Syndrome Society], while in U.K. a possessive term has been chosen – Down's Syndrome Association. Again, the variation is arbitrary, not governed by any definitive or obligatory principle.

This study also showed that there is a distinct difference in use of possessive form in two sides of the Atlantic – European publications still continue possessive eponyms, while north American counterpart use those overwhelmingly in the nonpossesive form. This difference existed in the past, and is continued with increasing preference towards nonpossesive form, although the uniformity is yet to be achieved in about 10–15% of articles. Recent PubMed search revealed compared to "Down syndrome," terms "Down's syndrome" and trisomy 21 yielded approximately 5% and 10% more articles, respectively. Therefore, missing those articles hampers effective literature search needing duplication of efforts.

Although suitable descriptive terminology based on patho-physiology (eg, trisomy 21 for Down syndrome) is generally favoured [5,8], such clear and precise alternatives may not be available for thousands of medical eponyms [13,15]. The advantages of an agreed eponym cannot be ignored as these are considered as labels or handles [5], and are useful substitutes for cumbersome, tongue-twister (eg, Susac syndrome for retinocochleocerebral vasculopathy) or offensive nomenclatures (eg, Hurler syndrome for gargoylism) [5]. A nonpossessive form is preferable as the "person behind the eponym" has no proprietary claim on it [3,5]. In some situations, a possessive form is unnecessary (eg, Williams syndrome), or even highly discouraged (after a patient's name eg, Hartnup disease) [5]. Whilst in other situations such as compound eponyms (eg, Klippel-Feil syndrome), and toponyms (naming after a place name, such as Australia antigen), a nonpossesive form is standard practice [3,5].

Eponyms have a long history in English, including medical English [3]. Therefore, the language of eponym should be a part of English language, which can accommodate both forms of eponym. On structural, semantic and historical grounds, nonpossesive medical eponyms find support, since the English language accommodates unmarked noun modifiers [3]. Further, as possessive eponym can blur (if not confuse) the grammatical and nongrammatical meanings of the genitive, it should be avoided [5,20]. As medical eponyms are primarily authorial (ie, the possessive noun designates a person who created or invented something) [3], a possessive form is often unwarranted, redundant, and confusing. As further progress occurs, some possessive eponyms have rather changed into derived adjectives: "Addison's crisis" has become "Addisonian crisis" [3].

In addition, the nonpossessive form that has no linguistic barrier [1], is also technically more efficient with fewer letters and no punctuation. Although one might feel initially awkward to drop "apostrophe s" from Kreb's cycle or Parkinson's disease, it would not be too difficult to master it with little persistence, as most of us spontaneously do with Apgar score, Bishop score or Cochrane review. The Council of Biology Editors (now the Council of Science Editors), a staunch advocate for harmonisation in stylistic standard has adopted an irrefutable posture: "It is recommended that the possessive form be eliminated altogether from eponymic terms so that they can be clearly differentiated from true possessives" [20,21]. In concurrence, American Medical Association (Manual of Style) and a major reference on medical eponyms have consistently omitted possessive form in eponymous terms long ago [15,22]. Furthermore, since 1993 the medical subject heading (MeSH) "Down's syndrome" has been replaced with "Down syndrome." "Reflecting the trend in current publications" one medical dictionary "has dropped the possessive form for eponymous term" [23], while the other still uses "inconsistent mixture of both forms" [9]. However, there remain few more barriers – many standard references including International Classification of Diseases continue to use both forms arbitrarily [9-13,24-26]. The International Committee of Medical Journal Editors has not provided any guideline on this issue, thereby allowing individual journal to choose either eponymous forms [27].

Despite the inconsistency, there is a gradual drift towards the nonpossessive form in nonmedical language such as newspapers, journals and other areas [3]. Amid all controversy, debate, and defence the medical eponyms live and thrive [1,2,15,28]. Therefore, it is important that a uniform nomenclature is followed worldwide. Cultivating uniformity is not a matter of ruthless attempt to bulldose its colourful diversity, but to reduce the burden of yet another source of confusion. The barriers created by possessive usage of medical eponyms are artificial and perhaps, unsustainable in the globalised world, and it should be removed by more sincere, logical and concerted efforts. Although "Down syndrome" has been taken as an illustration, the basic principles can be applied to thousands of eponyms in all walk of life [13,15,26]. Improved awareness of appropriate medical nomenclature is the joint responsibility of the authors, editors, reviewers, and publishers.

## Conclusion

Possessive form of eponyms is extant, but not extinct. Gradual decline of possessive form is evident in print and electronic publications. This decline is slow and sustained. As nonpossessive form is more efficient, simple and non-confusing, and has both linguistic support [3] and endorsement by major international organisations/groups [7,8,20-22], it has a wider acceptance. The present study suggests the need for more vigorous, systematic and sincere efforts in this direction worldwide.

## Competing interests

The authors declare that they have no competing interests.

## Authors' contributions

NJ conceived the idea of this study and provided the study design. All authors collected and analysed the data. NJ wrote the first draft, which was modified by SB and NA with critical inputs. All authors read and approved the final manuscript.

## Pre-publication history

The pre-publication history for this paper can be accessed here:

http://www.biomedcentral.com/1471-2288/9/18/prepub
